# Insect-associated bacterial communities across an anthropogenic landscape

**DOI:** 10.1128/msphere.00320-25

**Published:** 2025-08-28

**Authors:** Elina Hanhimäki, Susanna Linna, Camila Souza Beraldo, Mikael Englund, Uxue Rezola, Pedro Cardoso, Rose Thorogood, Marjo Saastamoinen, Anne Duplouy

**Affiliations:** 1 Organismal and Evolutionary Biology Research Program, Faculty of Biological and Environmental Sciences, University of Helsinki 3835, Helsinki, Finland; 2 Molecular and Integrative Biosciences Research Program, Faculty of Biological and Environmental Sciences, University of Helsinki 3835, Helsinki, Finland; 3 Finnish Museum of Natural History LUOMUS, University of Helsinki 3835, Helsinki, Finland; 4 Centre for Ecology, Evolution and Environmental Changes (CE3C), Laboratory for Integrative Biodiversity Research (LIBRe), CHANGE—Global Change and Sustainability Institute, Faculty of Sciences, University of Lisbon 37809, Lisbon, Portugal; 5 Helsinki Institute of Life Sciences (HiLIFE), University of Helsinki 3835, Helsinki, Finland; University of South Africa, Florida, Johannesburg, Gauteng, South Africa

**Keywords:** land-use, insects, microbiota, agricultural landscape, resilience

## Abstract

Anthropogenic activities induce drastic changes in land use that are at least partly responsible for the ongoing global patterns of macro-biodiversity erosion. These habitat changes also impact the fitness of the resilient species, through direct effects on diet and/or indirect environmental effects. Although microbial communities associated with species can crucially influence a diverse set of their host’s biological functions, studies on how microbial communities associated with wild species respond to habitat degradation remain scarce. We use metacommunities of two sympatric herbivorous insect species specialized in feeding on *Plantago lanceolata* that occurs across a network of natural meadows, pastures, and roadsides, as a model system to test the hypothesis that habitat degradation can also impact their associated microbial communities. The Glanville fritillary butterfly, *Melitaea cinxia*, and the weevil *Mecinus pascuorum* were sampled from local meadow habitats affected by various levels of disturbance (e.g., roads, agricultural fields, and buildings). The two species carry very distinct microbiota, either highly diverse and transient for the butterfly or dominated by a few resident bacterial symbionts for the weevil. Spatial characteristics of the focal habitat patch, namely the area of the meadow or the location of the meadow, explained the largest proportion of the variation in microbial community composition in both species, but these effects were significant only in the weevil. In contrast to our prediction, despite a minor increase in bacterial diversity along the gradient of habitat degradation, the overall composition of the microbiota was unchanged across habitats in both host species. Overall, local environmental characteristics other than habitat degradation explain the microbial associations in the two herbivorous insects of this system.

**IMPORTANCE:**

This research dives into the impact of habitat degradation on bacterial communities associated with wild herbivorous insect species, utilizing the ecologically relevant and well-characterized fragmented landscape of the Åland Islands, Finland. This study is crucial as habitat degradation driven by anthropogenic activities (i.e., land use change and habitat fragment size) poses a growing threat to global biodiversity. Indeed, as microbial partners play a pivotal role in the ecology and adaptation of their host species to their environment, there is a pressing need to comprehend how the host-associated microbial diversity responds to their host environmental changes to evaluate their contribution to the escalating patterns of biodiversity erosion globally. However, despite extensive research on the impact of habitat degradation on macro-species, the effects on microbial communities remain an understudied aspect of species ecology.

## INTRODUCTION

Deterministic processes drive assembly patterns of resident microbial communities, whereas neutral processes are more likely to result in the acquisition or loss of random and transient microbial species (1–3). In either case, alterations in the functional diversity of microbial communities can impact the development, digestion, fecundity, metabolism, immunity, health, and/or diverse other biological functions of their hosts (4, 5), but see (6, 7). Therefore, changes in microbiota may scale up to influence the resilience of macrobiota and the wide range of community interactions and ecosystem services that they provide (4, 5, 8). Nevertheless, although a large literature illustrates that habitat degradation is at least partly responsible for ongoing global patterns of erosion in macro-biodiversity (9–11), the impact of habitat degradation experienced by the host on their associated microbial biodiversity, resident and/or transient, has received little attention (12).

Habitat degradation is a multifaceted umbrella term, which is generally associated with negative impacts on biodiversity through decline of the local species richness or changes in community composition leading to biotic homogenization (13–16, and see review 17). For example, in a global analysis of the effect of land use change on the megadiverse group of the rove beetles, Méndez-Rojas et al. (18) showed that species density and richness decrease with increasing transformation of the beetles’ natural habitats into agricultural fields, whereas Uhl et al. (19) demonstrated that a decrease in habitat quantity across the landscape negatively affects the functional diversity of moths in Italian forest reserves. In contrast, fragmentation *per se* (i.e., breaking of the habitat into smaller fragments without total surface loss) has been argued to possibly promote local biodiversity due to the overall higher levels of habitat heterogeneity (20, 21). Although the debates on how qualitative and quantitative features of the habitat may affect macro-biodiversity are the theme of a large literature, little is known about how these features might affect the microbial communities associated with macro-species (i.e., host species). On one hand, under environmental change, we might anticipate that variation in the microbiota associated with species should reflect an active selection of beneficial microbes or purging of microbes detrimental to the host’s abilities to tolerate or resist stresses (22–26). However, it is also plausible that changes in host-associated microbial communities could simply reflect changes in the environmental microbiota, which the host encounters and collects at random through dispersing and feeding (27, 28).

A bedrock poor in nutrients but locally covered in rich sediments supports the different habitat types observed across the Åland Islands, a Finnish archipelago in the Baltic Sea (29). The islands feature areas covered by patchy managed forests (43% of land area), meadows, or peatlands, and they also experience agricultural (9% of land area) and rural pressures (29, 30). In this environment, a scattered network of over 4,400 geolocated meadows, or pastures, has been systematically surveyed since 1993 (31, 32). Each meadow differs from the next in many different quality aspects, including their surface area (i.e., the median meadow size is 0.06 hectares, Hanski et al. [33]), as well as the level of habitat degradation due to different human activities within the meadows themselves, and in the landscape surrounding each meadow (33, 34). Each meadow is characterized by the presence of the common ribwort plantain, *Plantago lanceolata* (31), and offers habitat for many species, including the Glanville fritillary butterfly *Melitaea cinxia* (Linnaeus, 1758) and the weevil *Mecinus pascuorum* (Gyllenhal, 1813), two specialized herbivores of *P. lanceolata* (31, 32). Consequently, the ecology and evolution of *P. lanceolata*, *M. cinxia*, and *M. pascuorum*, within the context of habitat loss and fragmentation observed in the Åland archipelago, have been the topic of numerous studies (35, 36).

The gut microbiota of *M. cinxia* caterpillars has been described as transient, with no effect on the butterflies’ larval development and survival (7). In this species, the microbial communities colonizing the host are possibly only representative of the host’s environment, but not completely similar to the microbiota of their host plants (37). These microbial studies are consistent with studies in other Lepidoptera species (6) and supplement the wealth of ecological, genetic, and genomic data already available for this butterfly species (38–40). In contrast, the details of the relationship between *M. pascuorum* weevils and their associated microbiota remain unknown. Nonetheless, as the literature shows that many weevil species carry a resident microbiota, dominated by a few bacterial symbionts with important mutualistic functions for their host fitness (41–43), it is likely that the Finnish *M. pascuorum* similarly evolves in association with symbiotic microbes. Overall, this system offers an opportunity to test whether habitat degradation, from meadows to agricultural and residential habitats, impacts the richness and composition of the microbial communities associated with these two sympatric insect species. However, because of their diverse relationship with their hosts, changes in either microbiota type might suggest different types of pressures on the hosts. In the context of host habitat degradation, changes in the transient microbiota associated with the butterfly *M. cinxia* could directly reflect changes in the environmental microbiota (7, 37); however, changes in the resident microbiota associated with the weevil *M. pascuorum* could rather suggest physiological, developmental, and/or immunological changes in the host (4, 44).

Here, we used metabarcoding techniques to independently characterize the bacterial microbiota associated with *M. cinxia* butterfly and with *M. pascuorum* weevil specimens, which were collected from meadows scattered across the Åland Islands. We suggest that bacterial community composition would differ between insects from small and large meadows and/or with the level of anthropogenic disturbances within the meadows or within the landscape, as could be expected in host species challenged by changes in the quality of their habitat or health. Furthermore, we expected that the quality of the host’s habitat would positively correlate with bacterial species richness in these insects, more so in the case of the transient microbiota of *M. cinxia* than in the possibly resident microbiota of *M. pascuorum*.

## MATERIALS AND METHODS

### Samples

**Fig 1:**
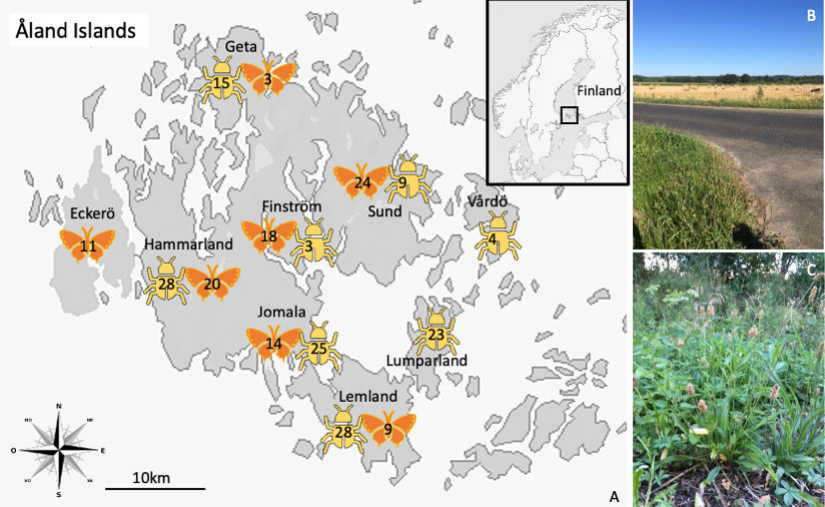
Sampling locations for *M. cinxia* caterpillars (orange symbols) and *M. pascuorum* weevils (yellow symbols) across the Åland Islands, Finland. Numbers on top of the symbols indicate the number of specimens collected within each sampling region and species. (Right top): a meadow within a degraded habitat type, (Bottom right): host plant *Plantago lanceolata* with flowers.

As described above, a network of over 4,400 geolocated meadows, or pastures (31, 32), which differ in many quality aspects, is scattered across the Åland islands (Fig. 1), an archipelago between the coasts of Finland and Sweden in the Baltic Sea. We collected specimens from two native insect species across this network of meadows. Caterpillars of the *M. cinxia* butterfly feed only on two host plants in the Åland Islands, *Plantago lanceolata* and *Veronica spicata* (31), and develop gregariously within overwintering silk nests (31) until their last larval instar, when they disperse to later pupate. The Åland population of the butterfly is surveyed on a yearly basis across the archipelago (31). Caterpillars selected for the present study were collected during one of those annual surveys and from silk nests built on *P. lanceolata* exclusively. About one-third of the wild collected *M. cinxia* caterpillars are naturally parasitized by the specialized parasitoid wasp, *Hyposoter horticola* (Gravenhorst, 1829) (45–47), and such parasitism slightly affects the host microbiota (37); however, detection of the parasitoid wasp at this stage of development is only possible through dissection or PCR (see below). Previous work in *M. cinxia* suggests the gut microbiota of this species is transient and highly variable (7, 37) and thus likely to be highly influenced by any change in the host habitat. On the other hand, the weevil *M. pascuorum*, which also specializes in feeding on *P. lanceolata,* feeds on the leaves of the host plant only as adults, whereas the larvae feed on the seeds of the host plant (48). The species overwinters at the adult and larval stages, produces two to three broods a year, and is only known to be attacked by parasitoid wasps at the larval stage (48). The microbiota of *M. pascuorum* has not been characterized; however, research on the microbiota of other weevil species could suggest a resident microbiota with mutualistic functions (41–43). All specimens were collected alive, individually placed in a tube labeled with a unique barcode, and stored at –20°C until further manipulation. There were no other fitness, health, or developmental trait measurements associated with any of the specimens.

To test the effect of habitat degradation on the microbiota of the two host species, we used 99 *M*. *cinxia* caterpillars collected in September 2012 for the purpose of another study (38), from 38 meadows in seven regions (i.e., Eckerö, Finström, Geta, Hammarland, Jomala, Lemland, and Sund). The surface area of these meadows varies between 89 and 49,463 m^2^ (33). Similarly, we collected 125 *M*. *pascuorum* adult specimens in June 2020, from 16 meadows in eight regions (i.e., Finström, Geta, Hammarland, Jomala, Lemland, Lumparland, Sund, and Vårdö) (Fig. 1). The surface area of these 16 meadows varies between 141 and 17,152 m^2^ (33). Each meadow was assigned two values of habitat degradation: (i) within the meadow and (ii) within the 10-meter buffer surrounding the meadow. Habitat degradation within the meadow captures direct aspects of the meadow quality, whereas the degradation within the 10 m buffer surrounding each meadow captures wider overall landscape degradation (34), which can affect the microclimatic conditions or colonization of the meadow. To measure habitat degradation, we calculated the proportion of surface area representing degraded versus natural land use in either the meadow or the 10 m buffer. The different land-use types were previously described by Hanski et al. (33) and represent 13 land-use categories, either associated with natural habitats (e.g., beaches, rocks, meadows, forests, and streams) or associated with habitat degradation due to anthropogenic activities (e.g., agricultural fields, roads, and built areas). The higher the proportion of land used for anthropogenic activities, the more degraded the habitat was considered. The land-use categories are based on the 2017 National Land Survey of Finland’s Topographic database and from the 2011 Multi-source National Forest Inventory (33). Since around 2010, the Åland Islands have faced modest shifts from traditional crop and livestock practices toward more organic farming (30). Similarly, although there is a gradual increase in housing development and road network upgrading, these infrastructure changes have principally occurred around the main town of Mariehamn (30), and less importantly in the regions sampled for the present study. The origin and metadata associated with each specimen, including the surface area and other characteristics of each meadow, can be found in Table S1 (see https://zenodo.org/records/15526656).

### Molecular work: DNA extraction and PCRs

All specimens were individually washed by plunging them in a 1× PBS bath for a few seconds to remove any contamination from the external environment and dissected under a sterile laminar flow cabinet to avoid any environmental contamination at the early stage of the sample preparation. As the cuticle from the skin and head might interfere with the first steps of the DNA extraction of *M. cinxia*, we pulled the head and the last larval segment of the specimens in opposite directions within a sterile 1× PBS solution, exposing the internal soft tissues. We further pressed the skin to empty it of all soft tissue material, cut the head away, and conducted the DNA extraction on all remaining soft tissues, including the gut content, collected in the dissection solution. Although this dissection protocol removes most of the external tissues of the caterpillars, we believe that both endophyte and at least some epiphyte microbial species associated with *M. cinxia* are included in the DNA extracts. In contrast, because of their small size, we used whole adult *M. pascuorum* weevils for individual DNA extractions. We used the same Qiagen DNeasy Blood and Tissue kit (Qiagen, Germany) following the manufacturer’s protocol for all extractions. Seven blank samples (sterile water samples; two per 96-well PCR plate) were similarly processed as controls for contamination across the dissection, extraction, PCR, and sequencing protocol. We tested for parasitism by the parasitoid wasp *H. horticola* within the *M. cinxia* caterpillars by amplifying a section of the protein-b11 from the wasp-integrated ichnovirus, using the primer pair 34 F-463R (45).

Prior to performing the PCRs, we tested the concentration of all DNA extracts using NanoDrop 2000 (Thermo Scientific) and diluted all samples to similar DNA concentrations within each species (i.e., 30 ng/µL for the caterpillars, and 15 ng/µL for the weevil). The ≈250 bp-long V5-V6 hypervariable region of the conserved bacterial 16S rRNA genes was amplified by PCR, using the 784F/1061R primers (49) modified with 50 bp-long adapters. We chose the V5-V6 region because it was previously successfully used for the characterization of *M. cinxia* microbiota and allowed checking for consistency between the studies (7, 37). The amplification was performed on a BioRad thermal cycler with a program of 5 min at 95°C, followed by a total of 40 cycles at 95°C for 1:40 s, 54.2°C for 1:45 s, 72°C for 1:40 s, and ending with the final extension at 72°C for 7 min. All specimens were amplified in duplicate using 3 µL of DNA extract. The duplicates were pooled and sequenced using a 300-Paired-End run on a MiSeq v.3. Illumina Sequencing platform, after dual indexing, at the Finnish Institute for Molecular Medicine, FIMM.

### Metabarcoding data processing

Sequence data were demultiplexed at the FIMM sequencing center. Paired-end reads were processed using QIIME2 v.2021.2 (50) separately for *M. cinxia* and *M. pascuorum* data sets but using the same pipeline. Primer sequences were removed using the QIIME2 plugin cutadapt v.2021.2.0. Read pairs were joined, quality-trimmed, and denoised using DADA2 v.2021.2.0 (for *M. cinxia*, forward read trimmed at 224 and reverse read trimmed at 135; for *M. pascuorum,* forward read trimmed at 245 and reverse read trimmed at 195). Taxonomy was assigned to the Silva 138 database (51) using the q2-feature-classifier plugin (52). Amplicon sequence variants (ASVs) that were not assigned to any bacterial phyla or that were classified as mitochondria or chloroplasts were removed. Next, we used the R v.4.0.4 package decontam v.1.10.0 (with the prevalence method using a threshold of 0.5) (53) to remove any procedure contaminant from the data based on the protocol controls (as described in 7, 37). We also removed all ASVs represented by no more than three reads across all samples for both species, after which a midpoint-rooted tree was produced.

### Statistical analyses

All analyses were done separately for the two species. All statistical tests were completed using QIIME2 v.2021.2 (50) and R v.4.0.4 (54) using the packages ggplot2 v.3.3.2 (55), vegan v.2.5.7 (56), and indicspecies v.1.7.12 (57). We tested our variables for normality using the Shapiro-Wilk normality test (58). We constructed principal coordinate analysis (PCoA) plots using phyloseq v.1.34.0 0 (59) to visualize any patterns of clustering throughout the habitat disturbance gradient.

#### Species diversity (alpha-diversity) and indicator species

To test the effect of the habitat degradation, within the 10 m buffer area surrounding each meadow or within the meadow itself, on the ASVs alpha-diversity of the microbiota of *M. pascuorum* or *M. cinxia*, we used linear models (with Shannon index) and negative binomial regression models (for observed ASVs count) including the level of habitat degradation (within the meadow or within a 10 m buffer) and the region from which the sample was collected as predictors to the models. Additionally, we added meadow size, and for *M. cinxia* only, parasitism by *H. horticola,* as additional explanatory variables to the models. The meadow size and parasitism status were, however, removed from the models to avoid overfitting, after the AIC model selection test showed adding them increased the complexity of the model without a significant improvement of its performance.

For each host species, we used the Indicspecies v.1.7.12 package (57) with the IndVal index and 9999 permutations to identify indicator species, or indicator ASVs, for habitat degradation. The IndVal index identifies species based on fidelity, or presence, in a particular group, and specificity, or relative abundance, to a particular group, such that the indicator ASVs are significantly more abundant in either group compared. As the indicspecies model does not accept continuous variables, we categorized the meadows into two distinct groups: the meadows surrounded by a 10 m area with no to little degradation (proportion of human activities < 0.25) and the meadows surrounded by a 10 m buffer area, of which more than a quarter is affected by human activities (proportion of human activities ≥ 0.25). This threshold was selected as it falls within the often-suggested critical range of suitable habitat cover (10%–30%) (60, 61) and offered a balanced sample size between the two categories in the present study.

#### Species community composition (beta-diversity) and predictability

To assess changes in the composition of microbiota of *M. cinxia* and *M. pascuorum*, we visualized the data using a principal coordinate analysis (PCoA) and calculated the permutational multivariate analyses of variance (adonis PERMANOVA with 999 permutations) using either Bray-Curtis (62) or Unweighted UniFrac (63) distances, and including the level of habitat degradation (within the meadow or within a 10-meter buffer), the meadow size, and the sampling region as explanatory variables using adonis2 function in vegan v.2.5.7 (56). To visualize the five most important ASVs affecting sample clustering, ordination biplots were assessed for both Bray-Curtis, which takes into account the abundance of each bacterial species, and unweighted UniFrac distances, which ignore abundance but take into account phylogenetic relationships between bacterial species. Parasitism status of the *M. cinxia* caterpillars, which showed no statistical effect on the model, was removed from the model to avoid overfitting. Additional details about the predicted functional composition of the microbiota of both host species are available in supplementary material S1 (see Fig. S1 and Table S2 at https://zenodo.org/records/15526656).

Furthermore, we examined how predictable, and thus distinguishable, the taxonomic profiles of the microbial communities associated with either *M. cinxia* or *M. pascuorum* specimens were throughout the gradient of habitat degradation, by using a random forest regression model with 100 trees in QIIME2 with plugin q2-sample-classifier (52). Random forest is a standard classification approach, which was shown to perform better classification accuracies for microbiome data than many other classification methods (64). The q2-sample-classifier plugin supports supervised machine learning tools for pattern recognition in microbiome data (52) by using the pathway abundance method and the EPA-ng placement tool (65), and it produces and plots a Bray-Curtis matrix with predicted functional pathways. We examined any patterns of specimen clustering in R using the adonis2 model (PERMANOVA with 999 permutations), including the level of habitat degradation within each meadow, or in the 10 m buffer surrounding each meadow, the meadow size, and the sampled location as explanatory variables.

## RESULTS

### Comparing the microbiota of the two host species

**Fig 2:**
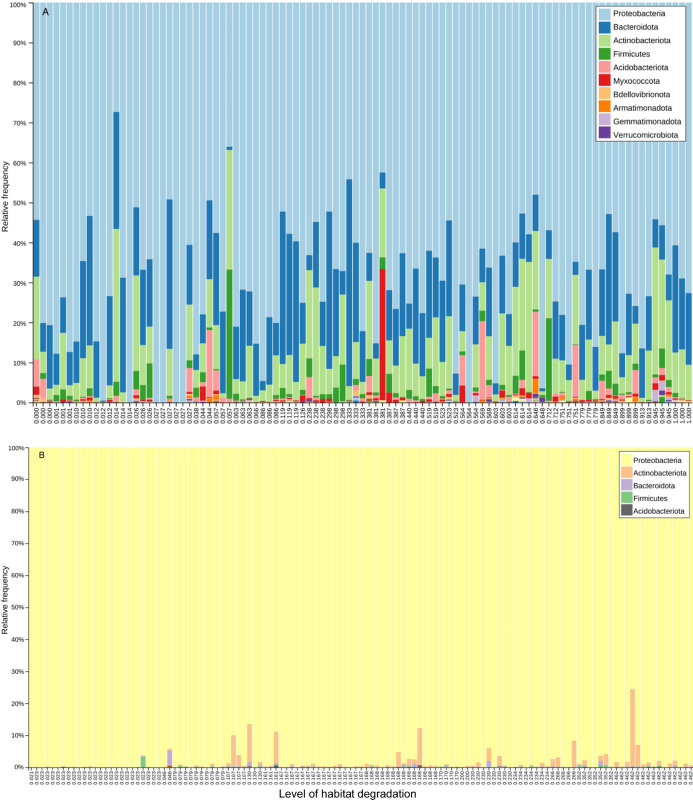
Relative abundance of bacterial phyla making up the microbiota colonizing (**A**) the gut of *M. cinxia* caterpillars, and (**B**) the full body of *M. pascuorum* weevils.

Although they share the same habitat and same host plant, caterpillars of the butterfly *M. cinxia* and adults of *M. pascuorum* weevil carry very distinct bacterial communities (Fig. 2). These microbial communities were thus treated separately to characterize patterns unique to each species.

For the butterfly microbial data set, the 99 samples provided a total of 2,553,069 sequences after the sequence cleaning and decontamination steps (8,741,919 original read pairs), which provided for 6,836 amplicon sequence variants (ASVs) with an average of 25,789 sequences and 373 ASVs per sample. This data set was normalized with rarefaction to 10,293 sequences, without leaving out any samples. The most abundant phyla associated with the gut of *M. cinxia* caterpillars were Proteobacteria, Bacteroidota, and Actinobacteriota (Fig. 2A), whereas the most abundant genera were *Yersinia*, *Sphingomonas,* and *Hymenobacter* (see Fig. S2A at https://zenodo.org/records/15526656).

For the weevil microbial data set, the 125 samples provided a total of 3,018,930 sequences after the sequence cleaning and decontamination step (14,849,912 original read pairs), which provided for 3,105 ASVs, with an average of 54,881 sequences and 2,227 ASVs per sample. This data set was rarefied to 28,936 sequences with no sample loss. Most bacterial species associated with *M. pascuorum* were from the Proteobacteria (Fig. 2B), including the most abundant genera that were three bacterial symbionts: *Rickettsia*, *Sodalis,* and *Wolbachia* (see Fig. S2B at https://zenodo.org/records/15526656).

**Fig 3:**
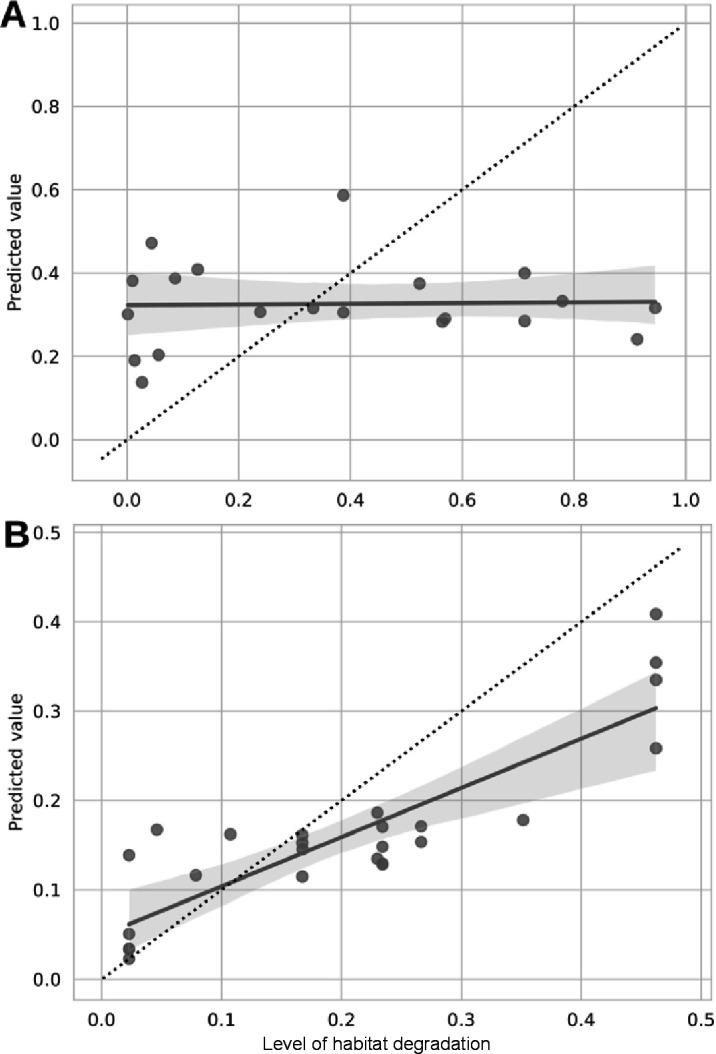
Scatterplots with linear regression lines with 95% confidence intervals (gray shading) presenting the accuracy results of the random forest q2-classifier for microbiota samples from (**A**) *M. cinxia* or (**B**) *M. pascuorum* across levels of habitat degradation. The plots show the correlation between true values (x-axis) and predicted values (y-axis) for each species. The dotted line represents the ideal 1:1 ratio between predicted and accurate values of microbiota composition.

Random forest regression accuracy was assessed based on the correlation between the true and predicted values for each specimen. As shown by Fig. 3, such regression model could not predict the microbiota of the butterfly *M. cinxia* (R2 = 0.001; mean squared error = 0.008; *P*-value = 0.908; Fig. 3A, Table S3 at https://zenodo.org/records/15526656) across our gradient of habitat degradation, but a similar model accurately predicted the bacterial profile of the weevil *M. pascuorum* across the gradient of habitat degradation (R2 = 0.748; mean squared error = 0.11; *P*-value = 2.47E-08; Fig. 3B, Table S3 at https://zenodo.org/records/15526656).

### Effects of sampling location had no effect on the nd habitat size on host species microbiota

For the butterfly *M. cinxia*, the sampling region (i.e., region with commune names) and focal meadow size had no effect on the observed ASV count, on the Hill-Shannon diversity of the microbiota (*P*-values > 0.05, Fig. S3 at https://zenodo.org/records/15526656), or on the composition of the microbiota of *M. cinxia* (*P*-value > 0.05, Fig. S4 at https://zenodo.org/records/15526656). Similarly, parasitism by the parasitoid wasp *H. horticola* also had no effect on the microbiota of *M. cinxia* (*P*-values > 0.05). The number of butterflies known to be parasitized was, however, relatively low (*N* = 23), and false-negative results are possible (37). These variables were later removed from the models as explained in the method section.

For the weevil *M. pascuorum*, the sampling location (i.e., region with commune names) and focal meadow size had no effect on the observed ASV count or on the Hill-Shannon diversity of the microbiota (*P*-values > 0.05, Fig. S3 at https://zenodo.org/records/15526656).

### Effects of habitat degradation on host species microbiota

#### Bacterial diversity (alpha-diversity) and indicator species

**Fig 4:**
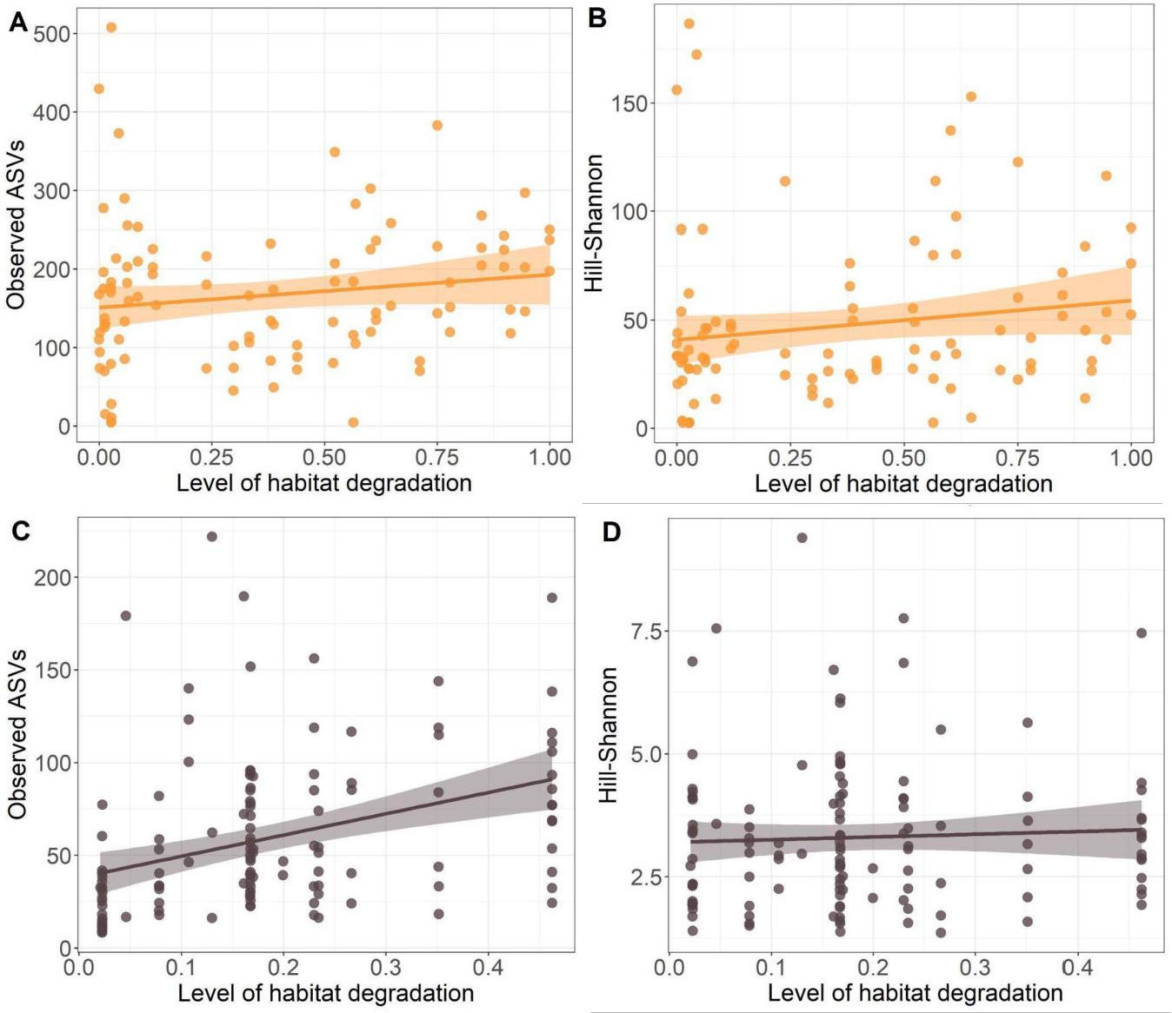
Variations in indices of alpha-diversity in the microbiota of the butterfly *M. cinxia* (orange) and the weevil *M. pascuorum* (gray) collected along a gradient of habitat degradation in a 10 m buffer area surrounding each meadow. (**A**) Observed ASVs and (**B**) Hill-Shannon index for *M. cinxia*, and (**C**) observed ASVs and (**D**) Hill-Shannon index for *M. pascuorum*. Colored areas along the lines indicate the 95% confidence intervals. *M. cinxia* was collected from a wider diversity of meadows across the Åland Islands than *M. pascuorum*.

For the *M. cinxia* caterpillars, the level of degradation within meadow or degradation in the 10 m buffer surrounding each meadow had no effect on the observed ASV count or on the Hill-Shannon diversity of the microbiota (*P*-values > 0.05; Fig. S3A and B; Fig. 4A and B, Table S4 at https://zenodo.org/records/15526656). The indicator species analysis found 12 bacterial ASVs that were significantly more often associated with specimens collected from meadows surrounded by degraded landscapes and 20 ASVs that were significantly more often associated with specimens from more natural surroundings (List of ASVs can be found in Table S5 at https://zenodo.org/records/15526656).

For the weevil *M. pascuorum*, the level of degradation within the meadow had no significant effect on the observed ASV count (*P*-value > 0.05; Fig. S3C at https://zenodo.org/records/15526656). However, the level of degradation within the meadow affected the Hill-Shannon estimates of alpha diversity (IRR = 11.92, 95% CI = 5.20–18.64, *P*-value = 0.001), with the Hill-Shannon index values significantly increasing with the level of degradation within the meadow (Fig. S3D; Table S4 at https://zenodo.org/records/15526656). The level of habitat degradation in the 10 m buffer surrounding each meadow had a significant effect on the observed ASVs (IRR = 11.87, 95% CI = 2.14–64.79, *P*-value = 0.01; Table S4 at https://zenodo.org/records/15526656), with the ASV count increasing with the level of habitat degradation in the surrounding landscape (Fig. 4C). However, the level of degradation in the 10 m buffer surrounding each meadow did not significantly affect the Hill-Shannon estimates of alpha-diversity (*P*-value > 0.05; Fig. 4D, Table S4 at https://zenodo.org/records/15526656). The indicator species analysis suggested 114 ASVs to be associated with more degraded habitats, and one ASV belonging to the *Sodalis* genus to be associated with more natural habitats (see Table S5 at https://zenodo.org/records/15526656).

#### Bacterial community composition (beta-diversity)

**Fig 5:**
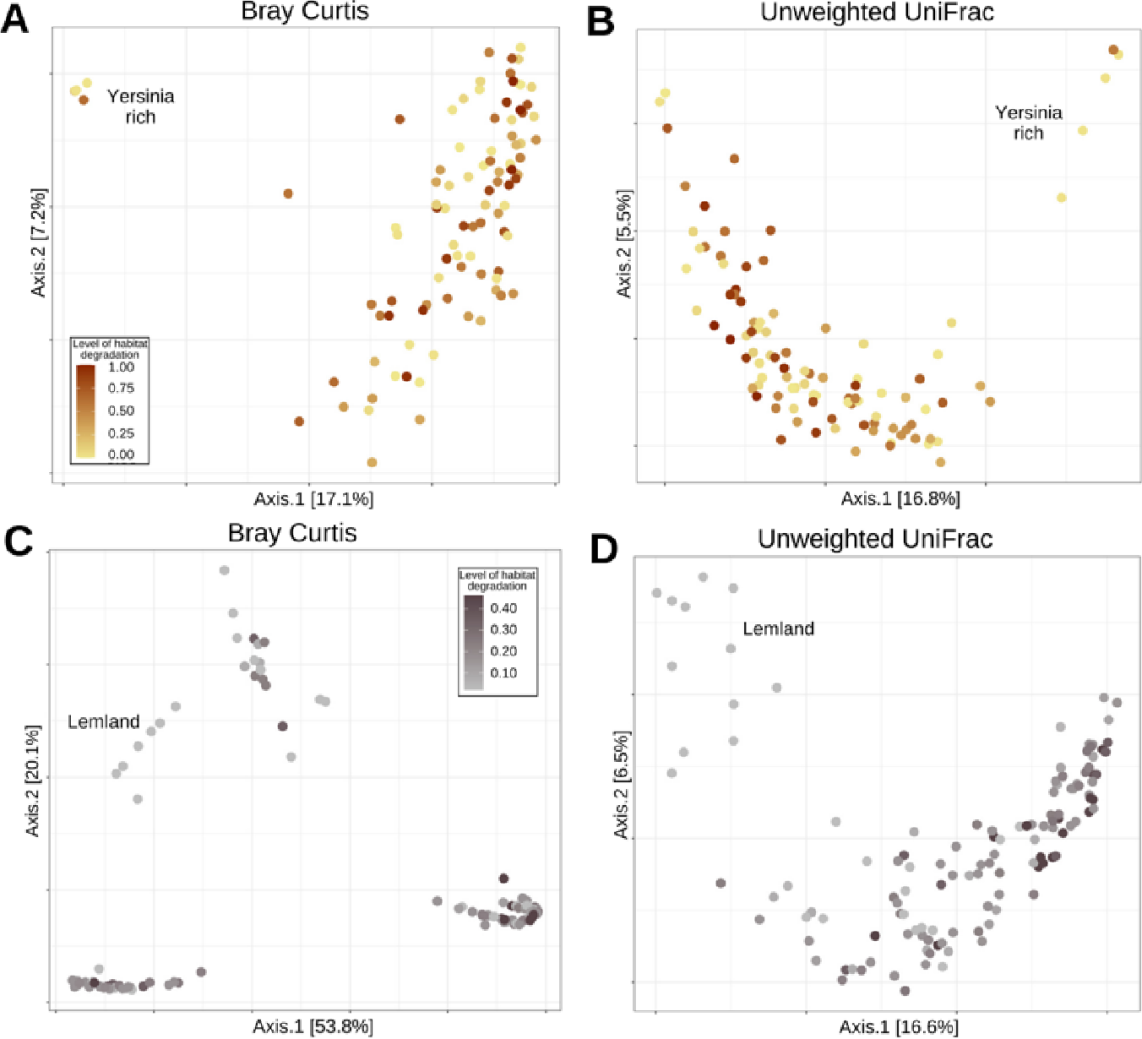
PCoA plots of the bacterial community composition (beta-diversity) of the microbiota associated with (left) *M. cinxia* and (right) *M. pascuorum*, using either Bray-Curtis (**A and C**) or Unweighted UniFrac (**B and D**) distances. The colors darken as the level of habitat degradation in the 10 m buffer area surrounding each meadow increases. Clear grouping of the “*Yersinia-rich” M. cinxia* specimens and “*Lemland” M. pascuorum* specimens is indicated in each panel.

In *M. cinxia*, the level of habitat degradation within meadows had no effect on bacterial community composition (*P*-values > 0.05; see Fig. S4A and B at https://zenodo.org/records/15526656). The influence of habitat degradation in the 10 m buffer surrounding the meadows on the community composition was not significant when tested using Bray-Curtis distance (Fig. 5A). We did find a significant impact of habitat degradation on microbiota composition when examined with Unweighted UniFrac distance (PERMANOVA: F = 2.156, R2 = 0.020, *P*-value = 0.009; Fig. 5B, Table S6 at https://zenodo.org/records/15526656). Most important bacterial ASVs affecting the PCoA biplot for Unweighted UniFrac were classified to the genus *Yersinia, Hymenobacter, Chrysobacterium, Sphigomonas,* and phylum Proteobacteria, and for Bray-Curtis, to genus *Yersinia, Wolbachia, Massilia, Chryseobacterium,* and the phylum Proteobacteria (see Fig. S5 at https://zenodo.org/records/15526656).

In *M. pascuorum*, the bacterial community composition was affected by the level of habitat degradation. The influence of habitat degradation within meadow (Bray-Curtis: PERMANOVA: F = 5.37; R2 = 0.034; *P*-value = 0.005; Unweighted UniFrac: PERMANOVA: F = 2.062; R2 = 0.015; *P*-value = 0.007; Fig. S4 C and D; Table S6 at https://zenodo.org/records/15526656) and in the 10 m buffer surrounding the meadows on the microbial community composition was significant with both Bray-Curtis and Unweighted UniFrac distance measures (Bray-Curtis: PERMANOVA, F = 10.499, R2 = 0.066, *P*-value = 0.001; Unweighted UniFrac: PERMANOVA, F = 6.760, R2 = 0.049, *P*-value = 0.001, Fig.5C and D; Table S6 at https://zenodo.org/records/15526656). Altogether, these results suggest that the weevils from more degraded meadows carried more homogenous microbiota. The PCoA plot illustrated that the microbiota of *M. pascuorum* weevils form four different groups. Most important ASVs affecting the PCoA biplots were classified to the genera *Sodalis* and *Rickettsia* and to the family Morganellaceae (see Fig. S6 at https://zenodo.org/records/15526656). The sampling location affected the community composition of the microbiota of *M. pascuorum* (Bray-Curtis: PERMANOVA, F = 3,459; R2 = 0.154; *P*-value = 0.001; Unweighted Unifrac: PERMANOVA, F = 1.761; R2 = 0.090; *P*-value = 0.001) (see Table S6 at https://zenodo.org/records/15526656), with specimens from Lemland (south-east of the main Åland island) showing a community profile enriched with *Sodalis sp*. (Fig. 5), a mutualistic bacterium known to provide threonine to other weevil species (66). Finally, the community composition of the microbiota associated with the weevils was significantly affected by the size of the focal meadow (Bray-Curtis: PERMANOVA, F = 3.480, R2 = 0.022, *P*-value = 0.019; Unweighted Unifrac: PERMANOVA, F = 2.487, R2 = 0.018, *P*-value = 0.002).

## DISCUSSION

The composition and species richness of microbial communities associated with organisms are shaped by a complex range of factors (1–3). Although habitat degradation has been reported to often cause severe stress to host species (16, 67), evidence of how species-associated microbiomes respond to those changes remains scarce. Our study evaluates how transient or resident bacterial communities associated with two sympatric insect species were affected by changes in their host shared habitat. Although we cannot generalize the findings of our study to all species, our data still provide a first comparison of the microbiota of these two herbivorous species and yield insights into the depth of habitat disturbance pressures on two local species with different microbiota characteristics and ecological roles.

Although both the caterpillars of the butterfly *M. cinxia* and the adult *M. pascuorum* weevils feed on the leaves of the same host plant species, *P. lanceolata* (31, 32), we show that their respective associated bacterial communities are very different, and our microbial community models could only predict the microbiota profile of the weevil host. *Melitaea cinxia* caterpillars carry a complex microbiota, rich in environmental bacterial species, which have no to little effects on the host’s biology (7, 37). Instead, the microbiota of the weevil adults quite strikingly converged into four distinct groups, each dominated by a bacterial species, described as maternally inherited symbionts in numerous other insects (i.e., two *Rickettsia, Sodalis,* and *Wolbachia*). Altogether, these results support the idea that the microbiota of *M. cinxia* butterflies is colonized by a transient microbiota subject to neutral processes, whereas that of the weevils is more predictable because resident and mutualistic and thus subject to deterministic processes (5, 41, 43, 44).

Resident microbiota are often dominated by inherited bacteria, such as *Wolbachia*, *Rickettsia*, or *Sodalis*, to only mention a few, which have been shown to manipulate their host reproductive system (42, 68, 69), support egg production (70), contribute to their host resistance against parasites (43, 71) or response to heat stress (26). The weevils from the Lemland region in the Åland Islands group together, as their microbiota is strikingly abundant with *Sodalis* bacteria, a bacterium with a specific mutualistic function in weevils. *Sodalis* are maternally inherited symbiotic bacteria, which provide weevils with amino acids necessary for the production of their thick protective cuticle after molting (41). Vigneron et al. (66) showed that in the cereal weevil *Sitophilus*, *Sodalis* densities increase within a week of the weevils molting but decrease once the new cuticle has hardened. Unfortunately, as our study does not include any trait and fitness measures for the host species, it remains unclear whether the age, developmental stage, sex, health, or any other fitness traits of the weevil host may vary with their associated microbial communities. All our weevil specimens were collected within four days of each other in mid-June, when the adult generation emerging from winter diapause reproduces, and the summer generation is going through the larval or nymphal stages within the *Plantago* seed capsules (32, 48). We suspected that all our weevil specimens must therefore belong to the winter generation; however, it is possible that the specimens collected in Lemland experienced a different microclimate that might have affected their developmental stage and allowed for the rapid molting and early emergence of specimens from the summer generation in this area. Future studies following changes in the microbial composition and density of obligate symbionts will inform us on the exact nature of the symbioses under different environmental and stress conditions.

Our study of the microbiota of both insects specializing on the same host plant species provided evidence that the characteristics of the host’s habitat can affect the local species communities (10, 72) even at the microbial level. Although the level of habitat degradation within the meadows and the size of the meadows only slightly affected the bacterial community composition associated with the weevil *M. pascuorum*, the level of landscape degradation (i.e., land-use changes in the 10 m buffer surrounding the meadows) led to a slight shift in the species composition of the bacterial microbiota associated with the two species tested. This pattern occurs in both species, although the insect species were collected in different years and from a different set of meadows. Although this could suggest a small, conserved effect of habitat degradation across species and through time, the Åland system is well known for experiencing interannual variations (35, 38), and only the study of microbial changes across habitats through several years could allow us to make temporal generalizations of these results. In contrast with our original expectations that increased habitat quality should correlate with increased associated microbial diversity in local host species, habitat degradation through land-use changes and reduction in habitat size do not however lead, in our system, to the loss of functional bacterial species diversity. Rather, we show that the bacterial species richness indices remain unchanged with habitat degradation in the butterfly *M. cinxia* and slightly but significantly increase in the weevil *M. pascuorum*. As microbial diversity losses are often more characteristic of stressed specimens (73), our results might suggest that despite habitat degradation, the Åland Islands are inhabited by an insect community with a microbiota that is little to not affected by the level of disturbances faced by these Finnish meadow habitats (74). Again, the generalization of such results remains difficult before robustly testing additional systems from different latitudes.

For many species, the composition of the microbiota, and especially that of the gut microbiota, is representative of their diet (75). Recently, Teng et al. (67) suggested that differences in the gut microbiota associated with populations from three rodent species were linked to changes in diets between populations. The animals from farmlands, where food diversity was high, hosted the most species-rich microbiota (67). In Åland, the adult weevils are specialist herbivores of *P. lanceolata* leaves (32), and although *M. cinxia* caterpillars can also feed on a second host plant, *Veronica spicata,* they were not recorded within our sampled meadows (35, 36). Thus, diversity within the microbiota of our specimens was unlikely due to drastic changes in diet between specimens, but such changes could still happen in species exhibiting more generalist diets than our two system species. Alternatively, the habitat degradation could lead to more subtle changes in the host plant, the host plant-associated microbiota, or in other elements of the insects’ environments (i.e., soil). The stronger effects observed for the weevil than for the butterfly may still support this hypothesis. In the Åland Islands, *Plantago-*associated bacterial communities are known to vary not only with the local road network (76) but also with the abundance of defensive iridoid glycoside compounds within the host plant (77), and with local soil microbial composition (78). The effect of the host plant diet was previously shown to have little effect on the microbiota of *M. cinxia* caterpillars (37), and thus, changes in the plant-associated microbiota are unlikely to severely affect the microbiota associated with the caterpillar. In contrast, the full body extractions of the weevil, instead of soft tissues DNA extractions in the butterfly, could indeed include more epiphytes with strong environmental interactions. Changes to local soil microbiota due to habitat degradation have been characterized worldwide (79, 80), and as the weevils crawl on soil surfaces when moving between plants and plant leaves (27), they could collect microbial species from their local soil. Additionally, adult weevils are generally not very mobile (81, 82), and *M. pascuorum* specimens are unlikely to disperse far or even outside their own meadow habitat, where they would encounter microbial species not representative of their local habitat. In contrast, although the caterpillars are also unlikely to disperse far, adult *M. cinxia* butterflies are well known for their dispersal ability between meadow habitats (35, 36). In *M. cinxia*, the effect of local habitat degradation on the microbiota could thus be diluted because the adult butterflies encounter diverse habitats during dispersal. Maternal effects on the microbiota of offspring were previously shown in insects (4, 6) and could thus confound the effect of local habitat degradation on the microbiota of the offspring. Minard et al. (37) have, however, shown that even *M. cinxia* caterpillars from the same family living on the same host plant individual carry very variable microbial communities, suggesting little maternal influence in this species. Nonetheless, future studies on the effect of habitat quality on local species-associated microbiota should integrate maternal effect and dispersal ability as potential key factors in the microbial heterogeneity across sampling sites.

The occurrence of diseases could also disturb the stability of host-associated microbial communities and allow the colonization of immune-challenged specimens by new microbes (83–85). The detection of *Yersinia* bacteria in a few *M. cinxia* caterpillars may suggest that our sampling included sick individuals upon collection in the field, as some *Yersinia* species are toxic to animals, including invertebrates (86, 87). In *M. cinxia* caterpillars, the clear shift toward a microbiota dominated by Enterobacteriaceae bacteria was previously observed in field-collected specimens (37), but not in caterpillars reared in the laboratory (7, 88). In contrast, the abundance of the bacterial taxon *Pseudomonas* in the transient gut microbiota of caterpillars from natural habitats may simply indicate that the bacterium is more abundant in these habitats, the cause of which remains unknown. *Pseudomonas* bacteria colonize a wide range of niches—some species are ubiquitous in soil, plant, and freshwater environments; and some are opportunistic pathogens of humans and other organisms (89), including insects (90).

Although habitat degradation is often associated with negative effects on macro-biodiversity level (16, 19, 67), the study of the impact of such anthropogenic changes on microbial communities associated with wild species (i.e., symbioses) remains scarce (12). Nonetheless, evaluating the responses of symbiotic microbial communities to habitat changes is crucial to predicting the vulnerability and resilience of macro-species to habitat degradation and any other anthropogenic changes. Our study contributes to bridging this gap by showing that changes in the shared habitat of two herbivorous species can indeed induce shifts in the composition of their associated microbial communities, sometimes even with an increase in bacterial species richness. This is in clear contradiction with our original expectations that degradation of the host habitat would be detrimental to their associated microbial diversity and functionality (67, 75). One possible explanation for these results is that habitat degradation in the Åland meadow network is not strong enough to lead to microbial erosion in the two insect species studied. Rather, it could offer opportunities for the insects to potentially experience more heterogeneous habitats, which support different sets of microbial species (as observed at the host species level in [19]). Although our system does not allow for testing more extreme levels of habitat degradation, it still suggests that remaining at least below a threshold of habitat destruction could support the functional stability of the microbiota associated with insects. Future studies, with larger sample sizes, will be able to characterize such a habitat degradation threshold from different systems and identify whether our results can be generalized across host-microbiota associations.

## Data Availability

The metabarcoding raw sequences for each sample, including the negative controls, are available from the NCBI database under the NCBI SRA project number PRJNA912103. Supplementary figures and tables are publicly available on Zenodo under the doi: 10.5281/zenodo.15526655.

## References

[1] Moran NA. 2007. Symbiosis as an adaptive process and source of phenotypic complexity. Proc Natl Acad Sci USA 104 Suppl 1:8627–8633. doi:10.1073/pnas.0611659104 17494762 PMC1876439

[2] Bénard A, Vavre F, Kremer N. 2020. Stress & symbiosis: heads or tails? Front Ecol Evol 8. doi:10.3389/fevo.2020.00167

[3] Unzueta-Martínez A, Welch H, Bowen JL. 2021. Determining the composition of resident and transient members of the oyster microbiome. Front Microbiol 12:828692. doi:10.3389/fmicb.2021.828692 35185836 PMC8847785

[4] Engel P, Moran NA. 2013. The gut microbiota of insects - diversity in structure and function. FEMS Microbiol Rev 37:699–735. doi:10.1111/1574-6976.12025 23692388

[5] Jing TZ, Qi FH, Wang ZY. 2020. Most dominant roles of insect gut bacteria: digestion, detoxification, or essential nutrient provision? Microbiome 8:38. doi:10.1186/s40168-020-00823-y 32178739 PMC7077154

[6] Hammer TJ, Janzen DH, Hallwachs W, Jaffe SP, Fierer N. 2017. Caterpillars lack a resident gut microbiome. Proc Natl Acad Sci USA 114:9641–9646. doi:10.1073/pnas.1707186114 28830993 PMC5594680

[7] Duplouy A, Minard G, Saastamoinen M. 2020. The gut bacterial community affects immunity but not metabolism in a specialist herbivorous butterfly. Ecol Evol 10:8755–8769. doi:10.1002/ece3.6573 32884655 PMC7452788

[8] Thorogood R, Mustonen V, Aleixo A, Aphalo PJ, Asiegbu FO, Cabeza M, Cairns J, Candolin U, Cardoso P, Eronen JT, Hällfors M, Hovatta I, Juslén A, Kovalchuk A, Kulmuni J, Kuula L, Mäkipää R, Ovaskainen O, Pesonen A-K, Primmer CR, Saastamoinen M, Schulman AH, Schulman L, Strona G, Vanhatalo J. 2023. Understanding and applying biological resilience, from genes to ecosystems. npj biodivers 2. doi:10.1038/s44185-023-00022-6 PMC11332022 39242840

[9] Borges PAV, Gabriel R, Fattorini S. 2020. Biodiversity erosion: causes and consequences, p 81–90. In Filho WL, Azul AM, Brandli L, Lange Salvia A, Wall T (ed), Life on Land, Encyclopedia of the UN Sustainable Development Goals

[10] Decaëns T, Martins MB, Feijoo A, Oszwald J, Dolédec S, Mathieu J, Arnaud de Sartre X, Bonilla D, Brown GG, Cuellar Criollo YA, et al.. 2018. Biodiversity loss along a gradient of deforestation in Amazonian agricultural landscapes. Conserv Biol 32:1380–1391. doi:10.1111/cobi.13206 30113727

[11] Horváth Z, Ptacnik R, Vad CF, Chase JM. 2019. Habitat loss over six decades accelerates regional and local biodiversity loss via changing landscape connectance. Ecol Lett 22:1019–1027. doi:10.1111/ele.13260 30932319 PMC6518933

[12] Hom EFY, Penn AS. 2021. Symbiosis and the anthropocene. Symbiosis 84:239–270. doi:10.1007/s13199-021-00794-0 34493891 PMC8414952

[13] Borgella R, Snow AA, Gavin TA. 2001. Species richness and pollen loads of hummingbirds using forest fragments in Southern Costa Rica . Biotropica 33:90–109. doi:10.1111/j.1744-7429.2001.tb00160.x

[14] Kumar A, O’Donnell S. 2007. Fragmentation and elevation effects on bird–army ant interactions in neotropical montane forest of Costa Rica. J Trop Ecol 23:581–590. doi:10.1017/S0266467407004270

[15] García-Martínez MÁ, Valenzuela-González JE, Escobar-Sarria F, López-Barrera F, Castaño-Meneses G. 2017. The surrounding landscape influences the diversity of leaf-litter ants in riparian cloud forest remnants. PLoS ONE 12:e0172464. doi:10.1371/journal.pone.0172464 28234948 PMC5325296

[16] Soh MCK, Mitchell NJ, Ridley AR, Butler CW, Puan CL, Peh KS-H. 2019. Impacts of habitat degradation on tropical montane biodiversity and ecosystem services: a systematic map for identifying future research priorities. Front For Glob Change 2:83. doi:10.3389/ffgc.2019.00083

[17] Cardoso P, Barton PS, Birkhofer K, Chichorro F, Deacon C, Fartmann T, Fukushima CS, Gaigher R, Habel JC, Hallmann CA, Hill MJ, Hochkirch A, Kwak ML, Mammola S, Ari Noriega J, Orfinger AB, Pedraza F, Pryke JS, Roque FO, Settele J, Simaika JP, Stork NE, Suhling F, Vorster C, Samways MJ. 2020. Scientists’ warning to humanity on insect extinctions. Biol Conserv 242:108426. doi:10.1016/j.biocon.2020.108426

[18] Méndez-Rojas DM, Cultid-Medina C, Escobar F. 2021. Influence of land use change on rove beetle diversity: a systematic review and global meta-analysis of a mega-diverse insect group. Ecol Indic 122:107239. doi:10.1016/j.ecolind.2020.107239

[19] Uhl B, Wölfling M, Fiedler K. 2021. Qualitative and quantitative loss of habitat at different spatial scales affects functional moth diversity. Front Ecol Evol 9:637371. doi:10.3389/fevo.2021.637371

[3] Fahrig L. 2003. Effects of habitat fragmentation on biodiversity. Annu Rev Ecol Evol Syst 34:487–515. doi:10.1146/annurev.ecolsys.34.011802.132419

[21] Riva F, Fahrig L. 2023. Landscape-scale habitat fragmentation is positively related to biodiversity, despite patch-scale ecosystem decay. Ecol Lett 26:268–277. doi:10.1111/ele.14145 36468190

[22] Fagundes CT, Amaral FA, Teixeira AL, Souza DG, Teixeira MM. 2012. Adapting to environmental stresses: the role of the microbiota in controlling innate immunity and behavioral responses. Immunol Rev 245:250–264. doi:10.1111/j.1600-065X.2011.01077.x 22168425

[23] Wernegreen JJ. 2012. Mutualism meltdown in insects: bacteria constrain thermal adaptation. Curr Opin Microbiol 15:255–262. doi:10.1016/j.mib.2012.02.001 22381679 PMC3590105

[24] Lemoine MM, Engl T, Kaltenpoth M. 2020. Microbial symbionts expanding or constraining abiotic niche space in insects. Curr Opin Insect Sci 39:14–20. doi:10.1016/j.cois.2020.01.003 32086000

[25] Houwenhuyse S, Stoks R, Mukherjee S, Decaestecker E. 2021. Locally adapted gut microbiomes mediate host stress tolerance. ISME J 15:2401–2414. doi:10.1038/s41396-021-00940-y 33658622 PMC8319338

[26] Tougeron K, Iltis C. 2022. Impact of heat stress on the fitness outcomes of symbiotic infection in aphids: a meta-analysis. Proc Biol Sci 289:20212660. doi:10.1098/rspb.2021.2660 35350854 PMC8965392

[27] Hannula SE, Zhu F, Heinen R, Bezemer TM. 2019. Foliar-feeding insects acquire microbiomes from the soil rather than the host plant. Nat Commun 10:1254. doi:10.1038/s41467-019-09284-w 30890706 PMC6425034

[28] Mason CJ, Hoover K, Felton GW. 2021. Effects of maize (Zea mays) genotypes and microbial sources in shaping fall armyworm (Spodoptera frugiperda) gut bacterial communities. Sci Rep 11:4429. doi:10.1038/s41598-021-83497-2 33627698 PMC7904771

[29] Haila Y, Järvinen O, Väisänen RA. 1980. Habitat distribution and species associations of land bird populations on the Åland Islands, SW Finland. Ann Zool Fenn 17:87–106.

[30] European Commission. 2022. Factsheet on 2014-2022 rural development programme for Åland Islands Finland. European Commission. https://agriculture.ec.europa.eu/system/files/2022-07/rdp-factsheet-finland-aland_en.pdf.

[31] Ojanen SP, Nieminen M, Meyke E, Pöyry J, Hanski I. 2013. Long-term metapopulation study of the Glanville fritillary butterfly (Melitaea cinxia): survey methods, data management, and long-term population trends. Ecol Evol 3:3713–3737. doi:10.1002/ece3.733 24198935 PMC3810870

[32] Nieminen M, Vikberg V. 2015. The insect community of Plantago lanceolata spikes in the Åland Islands, SWFinland. Entomol Fennica 26:30–52. doi:10.33338/ef.50914

[33] Hanski I, Schulz T, Wong SC, Ahola V, Ruokolainen A, Ojanen SP. 2017. Ecological and genetic basis of metapopulation persistence of the Glanville fritillary butterfly in fragmented landscapes. Nat Commun 8:14504. doi:10.1038/ncomms14504 28211463 PMC5321745

[34] Schulz T, Vanhatalo J, Saastamoinen M. 2020. Long‐term demographic surveys reveal a consistent relationship between average occupancy and abundance within local populations of a butterfly metapopulation. Ecography 43:306–317. doi:10.1111/ecog.04799

[35] Hanski IA. 2011. Eco-evolutionary spatial dynamics in the Glanville fritillary butterfly. Proc Natl Acad Sci USA 108:14397–14404. doi:10.1073/pnas.1110020108 21788506 PMC3167532

[36] Hanski I, Breuker CJ, Schöps K, Setchfield R, Nieminen M. 2002. Population history and life history influence the migration rate of female Glanville fritillary butterflies. OIKOS 98:87–97. doi:10.1034/j.1600-0706.2002.980109.x

[37] Minard G, Tikhonov G, Ovaskainen O, Saastamoinen M. 2019. The microbiome of the Melitaea cinxia butterfly shows marked variation but is only little explained by the traits of the butterfly or its host plant. Environ Microbiol 21:4253–4269. doi:10.1111/1462-2920.14786 31436012 PMC6900084

[38] Kahilainen A, van Nouhuys S, Schulz T, Saastamoinen M. 2018. Metapopulation dynamics in a changing climate: increasing spatial synchrony in weather conditions drives metapopulation synchrony of a butterfly inhabiting a fragmented landscape. Glob Chang Biol 24:4316–4329. doi:10.1111/gcb.14280 29682866 PMC6120548

[39] Smolander O-P, Blande D, Ahola V, Rastas P, Tanskanen J, Kammonen JI, Oostra V, Pellegrini L, Ikonen S, Dallas T, et al.. 2022. Improved chromosome-level genome assembly of the Glanville fritillary butterfly (Melitaea cinxia) integrating Pacific Biosciences long reads and a high-density linkage map. Gigascience 11:giab097. doi:10.1093/gigascience/giab097 35022701 PMC8756199

[40] van Bergen E, Dallas T, DiLeo MF, Kahilainen A, Mattila ALK, Luoto M, Saastamoinen M. 2020. The effect of summer drought on the predictability of local extinctions in a butterfly metapopulation. Conserv Biol 34:1503–1511. doi:10.1111/cobi.13515 32298001

[41] Anbutsu H, Moriyama M, Nikoh N, Hosokawa T, Futahashi R, Tanahashi M, Meng XY, Kuriwada T, Mori N, Oshima K, Hattori M, Fujie M, Satoh N, Maeda T, Shigenobu S, Koga R, Fukatsu T. 2017. Small genome symbiont underlies cuticle hardness in beetles. Proc Natl Acad Sci USA 114:E8382–E8391. doi:10.1073/pnas.1712857114 28923972 PMC5635926

[42] Hsiao C, Hsiao TH. 1985. Rickettsia as the cause of cytoplasmic incompatibility in the alfalfa weevil, Hypera postica. J Invertebr Pathol 45:244–246. doi:10.1016/0022-2011(85)90016-3

[43] White JA, Richards NK, Laugraud A, Saeed A, Curry MM, McNeill MR. 2015. Endosymbiotic candidates for parasitoid defense in exotic and native New Zealand weevils. Microb Ecol 70:274–286. doi:10.1007/s00248-014-0561-8 25613091

[44] Masson F, Moné Y, Vigneron A, Vallier A, Parisot N, Vincent-Monégat C, Balmand S, Carpentier MC, Zaidman-Rémy A, Heddi A. 2015. Weevil endosymbiont dynamics is associated with a clamping of immunity. BMC Genomics 16:819. doi:10.1186/s12864-015-2048-5 26482132 PMC4617454

[45] Duplouy A, Couchoux C, Hanski I, van Nouhuys S. 2015. Wolbachia infection in a natural parasitoid wasp population. PLoS ONE 10:e0134843. doi:10.1371/journal.pone.0134843 26244782 PMC4526672

[46] van Nouhuys S, Niemikapee S, Hanski I. 2012. Variation in a host-parasitoid interaction across independent populations. Insects 3:1236–1256. doi:10.3390/insects3041236 26466737 PMC4553574

[47] van Nouhuys S, Kohonen M, Duplouy A. 2016. Wolbachia increases the susceptibility of a parasitoid wasp to hyperparasitism. J Exp Biol 219:2984–2990. doi:10.1242/jeb.140699 27707863

[48] Nieminen M, Nouhuys S van. 2017. The roles of trophic interactions, competition and landscape in determining metacommunity structure of a seed-feeding weevil and its parasitoids. Ann Zool Fenn 54:83–95. doi:10.5735/086.054.0109

[49] Andersson AF, Lindberg M, Jakobsson H, Bäckhed F, Nyrén P, Engstrand L. 2008. Comparative analysis of human gut microbiota by barcoded pyrosequencing. PLoS One 3:e2836. doi:10.1371/journal.pone.0002836 18665274 PMC2475661

[50] Bolyen E, Rideout JR, Dillon MR, Bokulich NA, Abnet CC, Al-Ghalith GA, Alexander H, Alm EJ, Arumugam M, Asnicar F, et al.. 2019. Reproducible, interactive, scalable and extensible microbiome data science using QIIME 2. Nat Biotechnol 37:852–857. doi:10.1038/s41587-019-0209-9 31341288 PMC7015180

[51] Quast C, Pruesse E, Yilmaz P, Gerken J, Schweer T, Yarza P, Peplies J, Glöckner FO. 2013. The SILVA ribosomal RNA gene database project: improved data processing and web-based tools. Nucleic Acids Res 41:D590–6. doi:10.1093/nar/gks1219 23193283 PMC3531112

[52] Bokulich NA, Dillon MR, Bolyen E, Kaehler BD, Huttley GA, Caporaso JG. 2018. Q2-sample-classifier: machine-learning tools for microbiome classification and regression. J Open Res Softw 3:934. doi:10.21105/joss.00934 31552137 PMC6759219

[53] Davis NM, Proctor DM, Holmes SP, Relman DA, Callahan BJ. 2018. Simple statistical identification and removal of contaminant sequences in marker-gene and metagenomics data. Microbiome 6:226. doi:10.1186/s40168-018-0605-2 30558668 PMC6298009

[54] RCoreTeam. 2020. R: a language and environment for statistical computing, R foundation for statistical computing. Vienna, Austria. Available from: https://www.R-project.org

[55] Wickham H. 2016. ggplot2: elegant graphics for data analysis. Springer, Verlag New York.

[56] Oksanen J, Blanchet FG, Friendly M, Kindt R, Legendre P, McGlinn D. 2020. Vegan community ecology package version 2.5-7. https://cran.r-project.org/package=vegan.

[57] De Cáceres M, Legendre P. 2009. Associations between species and groups of sites: indices and statistical inference. Ecology 90:3566–3574. doi:10.1890/08-1823.1 20120823

[58] Shapiro SS, Wilk MB. 1965. An analysis of variance test for normality (complete samples). Biometrika 52:591–611. doi:10.1093/biomet/52.3-4.591

[59] McMurdie PJ, Holmes S. 2013. Phyloseq: an R package for reproducible interactive analysis and graphics of microbiome census data. PLoS One 8:e61217. doi:10.1371/journal.pone.0061217 23630581 PMC3632530

[60] Andrén H, Andren H. 1994. Effects of habitat fragmentation on birds and mammals in landscapes with different proportions of suitable habitat: a review. Oikos 71:355. doi:10.2307/3545823

[61] Swift TL, Hannon SJ. 2010. Critical thresholds associated with habitat loss: a review of the concepts, evidence, and applications. Biol Rev Camb Philos Soc 85:35–53. doi:10.1111/j.1469-185X.2009.00093.x 19930172

[62] Bray JR, Curtis JT. 1957. An ordination of the upland forest communities of Southern Wisconsin. Ecol Monogr 27:325–349. doi:10.2307/1942268

[63] Lozupone C, Lladser ME, Knights D, Stombaugh J, Knight R. 2011. UniFrac: an effective distance metric for microbial community comparison. ISME J 5:169–172. doi:10.1038/ismej.2010.133 20827291 PMC3105689

[64] Yu P, Shaw CA. 2014. An efficient algorithm for accurate computation of the Dirichlet-multinomial log-likelihood function. Bioinformatics 30:1547–1554. doi:10.1093/bioinformatics/btu079 24519380 PMC4081639

[65] Barbera P, Kozlov AM, Czech L, Morel B, Darriba D, Flouri T, Stamatakis A. 2019. EPA-ng: massively parallel evolutionary placement of genetic sequences. Syst Biol 68:365–369. doi:10.1093/sysbio/syy054 30165689 PMC6368480

[66] Vigneron A, Masson F, Vallier A, Balmand S, Rey M, Vincent-Monégat C, Aksoy E, Aubailly-Giraud E, Zaidman-Rémy A, Heddi A. 2014. Insects recycle endosymbionts when the benefit is over. Curr Biol 24:2267–2273. doi:10.1016/j.cub.2014.07.065 25242028

[67] Teng Y, Yang X, Li G, Zhu Y, Zhang Z. 2022. Habitats show more impacts than host species in shaping gut microbiota of sympatric rodent species in a fragmented forest. Front Microbiol 13:811990. doi:10.3389/fmicb.2022.811990 35197954 PMC8859092

[68] Giorgini M, Bernardo U, Monti MM, Nappo AG, Gebiola M. 2010. Rickettsia symbionts cause parthenogenetic reproduction in the parasitoid wasp Pnigalio soemius (Hymenoptera: Eulophidae). Appl Environ Microbiol 76:2589–2599. doi:10.1128/AEM.03154-09 20173065 PMC2849191

[69] Perlman SJ, Hunter MS, Zchori-Fein E. 2006. The emerging diversity of Rickettsia. Proc Biol Sci 273:2097–2106. doi:10.1098/rspb.2006.3541 16901827 PMC1635513

[70] Perotti MA, Clarke HK, Turner BD, Braig HR. 2006. Rickettsia as obligate and mycetomic bacteria. FASEB J 20:2372–2374. doi:10.1096/fj.06-5870fje 17012243

[71] Łukasik P, Guo H, van Asch M, Ferrari J, Godfray HCJ. 2013. Protection against a fungal pathogen conferred by the aphid facultative endosymbionts Rickettsia and Spiroplasma is expressed in multiple host genotypes and species and is not influenced by co-infection with another symbiont. J Evol Biol 26:2654–2661. doi:10.1111/jeb.12260 24118386

[72] Strona G, Lafferty KD. 2016. Environmental change makes robust ecological networks fragile. Nat Commun 7:12462. doi:10.1038/ncomms12462 27511722 PMC4987532

[73] Wei G, Lai Y, Wang G, Chen H, Li F, Wang S. 2017. Insect pathogenic fungus interacts with the gut microbiota to accelerate mosquito mortality. Proc Natl Acad Sci USA 114:5994–5999. doi:10.1073/pnas.1703546114 28533370 PMC5468619

[74] Lozupone CA, Stombaugh JI, Gordon JI, Jansson JK, Knight R. 2012. Diversity, stability and resilience of the human gut microbiota. Nature 489:220–230. doi:10.1038/nature11550 22972295 PMC3577372

[75] McManus N, Holmes SM, Louis EE, Johnson SE, Baden AL, Amato KR. 2021. The gut microbiome as an indicator of habitat disturbance in a critically endangered lemur. BMC Ecol Evol 21:222. doi:10.1186/s12862-021-01945-z 34915861 PMC8680155

[76] Numminen E, Laine AL. 2020. The spread of a wild plant pathogen is driven by the road network. PLoS Comput Biol 16:e1007703. doi:10.1371/journal.pcbi.1007703 32231370 PMC7108725

[77] Minard G, Kahilainen A, Biere A, Pakkanen H, Mappes J, Saastamoinen M. 2022. Complex plant quality-microbiota-population interactions modulate the response of a specialist herbivore to the defence of its host plant. Funct Ecol 36:2873–2888. doi:10.1111/1365-2435.14177 36632135 PMC9826300

[78] Mursinoff S, Tack AJM. 2017. Spatial variation in soil biota mediates plant adaptation to a foliar pathogen. New Phytol 214:644–654. doi:10.1111/nph.14402 28042886

[79] Li X, Jousset A, de Boer W, Carrión VJ, Zhang T, Wang X, Kuramae EE. 2019. Legacy of land use history determines reprogramming of plant physiology by soil microbiome. ISME J 13:738–751. doi:10.1038/s41396-018-0300-0 30368524 PMC6461838

[80] Kiesewetter KN, Afkhami ME. 2021. Microbiome-mediated effects of habitat fragmentation on native plant performance. New Phytol 232:1823–1838. doi:10.1111/nph.17595 34213774

[81] Lake EC, Hough-Goldstein J, Shropshire KJ, D’Amico V. 2011. Establishment and dispersal of the biological control weevil Rhinoncomimus latipes on mile-a-minute weed, Persicaria perfoliata. Biol Control 58:294–301. doi:10.1016/j.biocontrol.2011.05.005

[82] Smith L, Woods DM, Wibawa MI, Popescu V, Moran PJ, Villegas B, Pitcairn MJ, Hon C. 2021. Release and establishment of the weevil Mecinus janthiniformis for biological control of Dalmatian toadflax in Southern California. Biol Control 161:104633. doi:10.1016/j.biocontrol.2021.104633

[83] Fredensborg BL, Fossdal í Kálvalíð I, Johannesen TB, Stensvold CR, Nielsen HV, Kapel CMO. 2020. Parasites modulate the gut-microbiome in insects: a proof-of-concept study. PLoS ONE 15:e0227561. doi:10.1371/journal.pone.0227561 31935259 PMC6959588

[84] Zhang C, Derrien M, Levenez F, Brazeilles R, Ballal SA, Kim J, Degivry M-C, Quéré G, Garault P, van Hylckama Vlieg JET, Garrett WS, Doré J, Veiga P. 2016. Ecological robustness of the gut microbiota in response to ingestion of transient food-borne microbes. ISME J 10:2235–2245. doi:10.1038/ismej.2016.13 26953599 PMC4989305

[85] Abraham NM, Liu L, Jutras BL, Yadav AK, Narasimhan S, Gopalakrishnan V, Ansari JM, Jefferson KK, Cava F, Jacobs-Wagner C, Fikrig E. 2017. Pathogen-mediated manipulation of arthropod microbiota to promote infection. Proc Natl Acad Sci USA 114:E781–E790. doi:10.1073/pnas.1613422114 28096373 PMC5293115

[86] Bresolin G, Morgan JAW, Ilgen D, Scherer S, Fuchs TM. 2006. Low temperature-induced insecticidal activity of Yersinia enterocolitica. Mol Microbiol 59:503–512. doi:10.1111/j.1365-2958.2005.04916.x 16390445

[87] Springer K, Sänger PA, Moritz C, Felsl A, Rattei T, Fuchs TM. 2018. Insecticidal toxicity of Yersinia frederiksenii involves the novel enterotoxin YacT. Front Cell Infect Microbiol 8:392. doi:10.3389/fcimb.2018.00392 30488025 PMC6246891

[88] Duplouy A, Minard G, Lähteenaro M, Rytteri S, Saastamoinen M. 2018. Silk properties and overwinter survival in gregarious butterfly larvae. Ecol Evol 8:12443–12455. doi:10.1002/ece3.4595 30619557 PMC6309129

[89] Moore ERB, Tindall BJ, Martins Dos Santos VAP, Pieper DH, R J-L, Palleroni NJ. 2006. Nonmedical: *Pseudomonas*, p 646–703. In Dworkin M, Falkow S, Rosenberg E, Schleifer KH, Stackebrandt E (ed), The prokaryotes. Springer, New York, NY.

[90] Flury P, Aellen N, Ruffner B, Péchy-Tarr M, Fataar S, Metla Z, Dominguez-Ferreras A, Bloemberg G, Frey J, Goesmann A, Raaijmakers JM, Duffy B, Höfte M, Blom J, Smits THM, Keel C, Maurhofer M. 2016. Insect pathogenicity in plant-beneficial pseudomonads: phylogenetic distribution and comparative genomics. ISME J 10:2527–2542. doi:10.1038/ismej.2016.5 26894448 PMC5030700

